# Curriculum hierarchies as conversion factors: career guidance, capability, and inequality in South African schools: a conceptual review

**DOI:** 10.3389/fpsyg.2026.1870160

**Published:** 2026-07-23

**Authors:** Nthabeleng Innocentia Mdhluli, Renaldo Naude, Sekgoma Elsie Ramasodi, Busisiwe Mahlangu, Charmaine Murabi, Freddy Sedumedi Kau, Azwihangwisi Singo

**Affiliations:** Department of Industrial and Organisational Psychology, University of South Africa, Pretoria, South Africa

**Keywords:** capability approach, career guidance, curriculum hierarchy, Life Orientation, South Africa

## Abstract

In examination-centered education systems, career guidance frequently reproduces inequality rather than reducing it. South Africa illustrates this pattern. The Curriculum and Assessment Policy Statement (CAPS) requires career guidance through the required course Life Orientation (LO). However, LO has a structurally marginal position within CAPS because it is not evaluated externally and is not included in the point calculations used by the majority of South African universities for admission. The result is a compliance gap between policy intent and the institutional conditions required to realize that policy. This conceptual review develops a theoretical framework that explains how curriculum governance produces and reproduces career guidance inequality. Its central argument is that curriculum classification and framing rules act as structural-environmental conversion conditions which mediate whether learners can convert formal access to career guidance into substantive vocational capability. The framework integrates the sociology of knowledge with the Capability Approach. It draws on classification, framing, and recontextualization to explain curriculum governance, and on conversion factor theory to explain how institutional conditions shape capability development. Existing research on South African career guidance is used illustratively to ground four conceptual propositions. The manuscript’s principal theoretical contribution is the conceptualization of curriculum classification and framing rules as a form of structural-environmental conversion condition, an institutional configuration that mediates the transformation of career guidance resources into substantive vocational capability. The review is conceptual rather than systematic in design. Career guidance inequality is not primarily a deficit of learner aspiration. It is a consequence of institutionally patterned conversion conditions. The framework carries implication for any examination-centered system in which subjects are differentially governed and resourced.

## 1 Introduction

Why do universal education policies so often produce systematically unequal outcomes? In South Africa, part of the answer lies in the framework of the curriculum itself. Curricula are not neutral inventories of knowledge. They are governance structures whose internal hierarchies determine which subjects attract sustained institutional investment, professional infrastructure, and pedagogic attention, and which are quietly left to operate on the margins (Bernstein, 2000; Hoadley, 2017).

The Curriculum and Assessment Policy Statement (CAPS) mandates career guidance for all secondary learners through the compulsory subject Life Orientation (LO). In practice, however, a clear compliance gap exists between policy intent and what learners actually receive. This review defines that gap as the systematic divergence between what CAPS mandates for career guidance and the institutional conditions required to deliver it. Learners in well-resourced schools tend to receive sustained, individualized career coaching. Learners in marginalized settings encounter only sporadic information with little follow-through (Albien and Naidoo, 2017; Jonck and Swanepoel, 2019). The variation is not random. It follows the curriculum’s own hierarchy of subjects and their governance status.

Despite three decades of post-apartheid reform, the South African school system remains deeply bifurcated. South African public schools are grouped into five quintiles (Q1 to Q5) on the basis of the socio-economic composition of the communities they serve. Schools in Quintiles 1–3 are designated “no-fee” schools and constitute approximately sixty per cent of public schools, serving the majority of learners (Department of Basic Education [DBE], 2019; Equal Education, 2021). Schools in Quintiles 4 and 5 are permitted to charge fees and serve historically more advantaged communities. School quintiles still serve as functional proxies for inherited socio-economic advantage, reflecting allocation patterns whose effects continue to structure provision in the present (Spaull and Jansen, 2019; Van der Berg, 2007). The scale of the challenge is significant. In the first quarter of 2025, approximately 46 per cent of South Africans aged 15–34 were unemployed (Statistics South Africa, 2025), and among those aged 15–24 the rate rose to 62 per cent, underscoring the practical stakes of the transition from schooling to work. Within this landscape, LO occupies a precarious position. It is compulsory, yet internally assessed [Department of Basic Education [DBE], 2011]. It is not externally examined. It is also excluded from the Admission Points Score calculations used by most South African universities (Diale et al., 2014). LO is therefore present in every school timetable but absent from the structures that confer institutional weight on a subject. This configuration produces what can usefully be described as a self-reinforcing cycle of disinvestment. In examination-centered systems, much of the institutional pressure that drives teacher development, subject advisor appointment, and regular inspection is generated by external accountability requirements (Hooley et al., 2013; Sultana, 2011). Subjects that escape external scrutiny tend, over time, to attract fewer resources and lower professional priority (Hoadley, 2017; OECD, 2004). LO’s structural position therefore directly shapes the unequal distribution of career guidance infrastructure across school quintiles. The mechanism is not malicious; it is governance-driven.

Two strands of scholarship have engaged with this field, but largely in parallel. Across this scholarship, three consistent patterns emerge. First, LO is documented as institutionally marginal and unevenly delivered (Diale et al., 2014; Maree, 2020; Magano, 2011). Second, the psychometric instruments most widely used in South African career guidance have been developed within cultural frames that do not consistently match the lives and labor market realities of the learners they are applied to Stead (2004), Stead and Watson (2006), and Watson et al. (2022). Third, collectively these two lines of scholarship point to a common underlying condition: the fragmentation of institutional infrastructure that would otherwise enable career guidance to convert formal enrollment into substantive vocational capability. What remains underdeveloped is the analytic bridge between them. How does the weak classification and internal assessment of LO translate into the fragmented professional infrastructure that shapes assessment practice? And how does that fragmentation, in turn, constrain learners’ capacity to convert career-related resources into informed choices? Underinvestment in career guidance, it should be noted, is not unique to South Africa. Comparative evidence indicates that examination-centered systems internationally tend to deprioritize guidance provision (Hooley et al., 2013; OECD, 2004; Sultana, 2011). To address the South African case, however, requires a framework that integrates curriculum sociology with the evaluative apparatus of capability theory.

Bernstein’s (2000) sociology of knowledge offers a precise account of why some subjects attract institutional investment while others are sidelined. In Bernstein’s framework, subjects with strong classification and strong framing of pedagogic communication are absorbed into the national accountability system. Strong classification refers to clearly insulated and well-bounded knowledge domains. The integration of such subjects into national governance occurs through two interrelated institutional fields. The first is the Official Recontextualizing Field (ORF), which includes state policy, examination governance, and external inspection. The second is the Pedagogic Recontextualizing Field (PRF), which is the institutional space in which policy mandates are translated into teacher education, subject advisory services, and curriculum materials (Bernstein, 2000, pp. 33–37). Subjects in these fields tend to have strong professional infrastructure, including regular inspection, dedicated subject advisors, specialist initial teacher education, and ongoing professional development (Hoadley, 2017; Muller, 2000). Subjects with weak classification and weak framing operate under quite different conditions. LO is an obvious example. Its knowledge boundaries are permeable and its pedagogic control broad, leaving it structurally peripheral to the PRF and, by extension, to the institutional flows of investment that follow.

In this phase, it is necessary to provide a theoretical clarification. The marginalization of LO is not, in Bernstein’s (2000) vocabulary, an expression of evaluation rules. Evaluation rules are epistemic criteria that determine what counts as a legitimate or excellent realization of knowledge within a subject. The challenge with LO is different in that it falls outside the investment logic of high-stakes curriculum governance due to poor classification and framing, regardless of how its internal evaluation is configured. Empirical research in South Africa supports this interpretation, demonstrating that subjects with poor classification consistently receive less infrastructural investment (Hoadley, 2017; Muller, 2000).

The second framework is provided by Sen’s (1999) Capability Approach. Robeyns (2005) puts this framework into practice by drawing the well-known distinction between resources, capabilities, and functions. Tests, brochures, counselors, and advisory periods are all examples of resources. Capabilities are the substantive opportunities those resources make possible, that is, the real freedom to pursue a valued life path. The accomplishments that are eventually realized are called functionings. The idea of conversion factors, which characterizes the structural, social, and personal circumstances that determine whether a given resource is truly usable by a specific person in a specific setting, connects these three categories.

Sen’s (1999) typology is expanded upon by Robeyns (2005) into three conversion factor categories: environmental, social, and personal. Robeyns (2005) demonstrates that the conversion of resources into capabilities can be systematically restricted by each category. The present review proposes a modest extension of this typology. In order to clarify the institutional and governance aspects of curriculum organization, it treats Robeyns’s environmental category as “structural-environmental.” Walker and Unterhalter (2007) anticipate this shift within education, demonstrating that institutional arrangements within schools are primary determinants of whether learners are able to develop and exercise meaningful capabilities. Nussbaum (2000) 2011 goes one step further and contends that, independent of the learner’s personal traits or goals, institutional provision failures amount to capability deprivation in and of themselves.

The manuscript positions itself at the intersection of four scholarly traditions, namely Bernsteinian curriculum sociology, capability-approach educational scholarship, international career guidance policy studies, and South African career psychology. Its distinctive contribution is the conceptualization of curriculum classification and framing rules as a form of structural-environmental conversion condition. To the best of the authors’ knowledge, this configuration has not yet been explicitly named in the scholarship. Anchoring Bernstein’s (2000) governance logic within Robeyns’ (2005) conversion factor typology repositions the analytic focus, shifting it from delivery modality, the customary preoccupation of school-based career guidance research, to curriculum governance architecture as a primary determinant of career guidance inequality. Existing South African research on career guidance suggests a consistent pattern. Where LO is poorly classified and framed, the PRF supporting the subject remains fragmented: teacher training is uneven, subject advisory services are thinly distributed, and psychometric tools are frequently deployed without the interpretive infrastructure needed for valid score application (Stead and Watson, 2006; Watson et al., 2022). The phenomenon is most noticeable when learner-to-counselor ratios are unmanageable, practitioner training is inadequate, or feedback time is inadequate. These conditions are specifically identified as prerequisites for legitimate score use in Kane’s (2013) interpretive argument model. The implication is straightforward but significant in a sense that fragmentation shapes learners’ capabilities, influencing decision-making clarity and the ability to translate aspirations into feasible post-school pathways.

Three questions are addressed in the review:

1. How do curriculum classification and framing rules shape institutional provision of career guidance in South African secondary schools?
2. How does institutional infrastructure mediate the contextual alignment and interpretive validity of psychometric career assessment practices?
3. How do these institutional conditions pattern learners’ educational preparedness, specifically agency, transition readiness, and sense of future possibility?

This study falls under developmental psychology because it examines the institutional conditions that shape adolescents’ ability to form, evaluate, and act on future-oriented career intentions. The formation of a coherent occupational identity is widely recognized as one of the primary developmental tasks of secondary school (Erikson, 1968; Skorikov and Vondracek, 2011). The institutional conditions that scaffold or constrain this process are therefore a legitimate, indeed unavoidable, concern of the field. By integrating Bernstein’s (2000) sociology of knowledge with Sen’s (1999) Capability Approach, this review offers a theoretically grounded account of how curriculum hierarchies produce unequal career outcomes. The aim is to move beyond descriptive accounts of disparity and to specify the structural mechanisms through which inequality is institutionally reproduced. The framework is anchored in South Africa, but the dynamics it describes are characteristic of examination-centered systems globally. Parallel patterns are visible in Sub-Saharan African studies on youth career choice (Akosah-Twumasi et al., 2018) and in comparative analyses across OECD member and partner countries (OECD, 2004; Watts and Fretwell, 2004).

## 2 Review approach

This paper is a conceptual review in the sense elaborated by Jaakkola (2020), Hulland (2020), and Cornelissen (2017). Its purpose is to develop a theoretical framework and to derive falsifiable propositions for later empirical testing, rather than to characterize the state of an evidence base. The design differs by intent from a systematic review, a scoping review, and a narrative synthesis, and this section makes the design explicit.

The review engages scholarship across four theoretical traditions: Bernsteinian curriculum sociology, capability-approach educational scholarship, international career guidance policy studies, and South African career psychology. Its geographic focus is South African secondary schooling, situated in international comparative context through Sub-Saharan African, OECD, and Commonwealth policy literatures. The temporal scope is deliberately wide, engaging the foundational theoretical works from the 1990s and 2000s alongside contemporary empirical research, including recent South African career guidance studies from 2017 to 2024.

Sources were selected according to five principles. First, sources had to speak directly to at least one of the four propositions or to their integration. Second, South African sources were prioritized where they provided proximal illustration of the conditions the framework theorizes. Third, sources across qualitative, quantitative, and mixed-methods traditions were included where each contributed a distinctive analytic angle. Fourth, sources were verified against original publication records and required a functional publication trail. Fifth, international sources were included to establish the transferability of the framework beyond the South African setting.

Research is drawn upon illustratively rather than comprehensively. Studies are engaged to ground and exemplify the theoretical propositions, not to demonstrate the state of the evidence. The four conceptual propositions in Section 4 were derived from the integrated Bernstein–capability framework by a process of theoretical elaboration, in which the mechanisms specified by Bernstein’s classification–framing distinction were mapped onto the resource–capability conversion process specified by Robeyns’s typology. Where a proposition is supported by an empirical study, the study is cited to illustrate the proposition; the proposition itself derives from the theoretical structure.

The review’s contribution should be evaluated against the criteria appropriate to conceptual work: theoretical coherence, generative capacity, and empirical testability (Cornelissen, 2017; Jaakkola, 2020). Sections 4 and 5 speak to coherence, Section 5 speaks to generative capacity through the Testable Propositions Overview and the Operational Indicators Table, and Section 8 states the empirical limitations that a subsequent program of validation would need to address.

## 3 Theoretical framework

### 3.1 Bernstein’s sociology of knowledge

F Bernstein’s (2000) sociology of education provides the structural analytic for this review. It clarifies the reason LO, despite its formal curriculum status, is unable to attract the institutional investment that would make career guidance effective. Classification, framing, and the recontextualizing fields are three of Bernstein’s ideas that are essential to the analysis.

The degree to which the curriculum’s knowledge domains are separated is referred to as classification (Bernstein, 2000, pp. 6–9). Subjects with strong classifications, like history, mathematics, and the physical sciences, have distinct, well-bounded knowledge structures. LO is a prime example of a weakly classified subject with permeable boundaries and a tendency toward integrationist orientations across multiple domains. The distinction does not constitute a qualitative assessment of pedagogical value. It explains the structural reasoning behind curriculum governance’s distribution of institutional resources. Muller (2000) and Maton (2014), working from somewhat different theoretical positions, both demonstrate that strongly bounded knowledge domains attract greater institutional investment as they align more naturally with the accountability metrics that examination governance requires.

Framing refers to control over the selection, sequencing, pacing, and criteria of pedagogic communication (Bernstein, 2000, pp. 12–16). Subjects governed by strictly defined external curricula and standardized assessment criteria are characterized by strong framing. Weak framing, characteristic of LO, produces diffuse pedagogic criteria and low external accountability. Taken together, weak classification and weak framing place LO at the margins of the Official Recontextualizing Field, the state-level space of examination governance and curriculum inspection. They also place it at the margin of the Pedagogic Recontextualizing Field, the institutional space in which policy mandates are translated into teacher education, subject advisory provision, and curriculum materials (Bernstein, 2000, pp. 33–37).

A clarification is required at this point due to a recurring misunderstanding in the field. Bernstein’s evaluation rules form the third element of his classification–framing–evaluation triad. They specify the epistemic criteria that determine what counts as a legitimate or excellent realization of knowledge within a subject. The structural marginalization of LO is not solely due to miscalibrated evaluation rules. It is a function of weak classification and weak framing, which together position the subject outside the accountability circuits that generate institutional investment. When LO’s internal assessment status is misinterpreted as an example of Bernstein’s evaluation rules, the analytic validity of his framework is significantly undermined (Hoadley, 2017; Muller, 2000). This line of reasoning is expanded upon by Legitimation Code Theory (Maton, 2014). It demonstrates how the semantic and structural properties of knowledge domains shape the institutional legitimacy and resource investment they attract within school systems.

### 3.2 The capability approach

Sen’s (1999) Capability Approach provides the evaluative framework for understanding what curriculum marginalization means at the level of learners. Sen distinguishes between commodities (resources), capabilities (real opportunities), and functionings (actual achievements). The distinction reframes the question of career guidance inequality in an important way. The question is no longer simply whether learners received resources, but whether they were genuinely able to convert those resources into vocational freedom. The shift is consequential. Two learners may receive formally identical career guidance resources and yet emerge with markedly different capabilities, because the conversion conditions surrounding them differ. Conversion conditions, in this sense, are the institutional scaffolding that mediates the move from resource to capability. Robeyns (2005) provides the standard elaboration of conversion factors within the Capability Approach. Three levels are identified by the author. Personal conversion factors include age, disability, and cognitive characteristics. Social conversion factors include norms, gender relations, and broader social hierarchies. Environmental conversion factors include geography, infrastructure, and institutional context. The current review expands Robeyns’ environmental category to “structural-environmental” in order to better understand the governance dimensions of curriculum organization. Specifically, this elaboration foregrounds the classification and framing rules that determine the institutional investment a subject attracts and, by extension, the conversion infrastructure available to learners. The resulting typology with curriculum-specific elaborations is presented in Table 1.

**TABLE 1:** Conversion factor typology applied to career guidance in South African schools.

Conversion factor level	Robeyns (2005) category	Curriculum governance elaboration
Personal	Personal	Learner’s prior academic preparation, language of instruction proficiency, and individual resilience resources
Social	Social	Cultural alignment of career assessment instruments; community norms around occupational aspiration; parental labor market knowledge networks
Structural-environmental	Environmental (elaborated)	Curriculum classification and framing rules; PRF investment levels; subject advisor ratios; teacher specialist training rates; learner-to-counselor ratios across quintiles

“Structural-environmental” elaborates Robeyns’s (2005) “environmental” category to foreground institutional governance dimensions of curriculum organization. The typology is applied analytically. Specific threshold values for the indicators listed require empirical derivation through future research.

Walker and Unterhalter (2007) demonstrate that institutional arrangements within schools are primary determinants of whether learners develop and exercise meaningful capabilities. Their argument applies directly to the career guidance context. Formal enrollment in LO does not, in itself, produce vocational capability. Without specialist-trained teachers, adequate contact time, and sustained advisory continuity, the formal entitlement remains nominal. Nussbaum (2000) 2011 strengthens this position considerably. Nussbaum (2000) 2011 contends that failures in institutional provision are a form of capability deprivation, rather than a personal deficit on the part of the learner.

### 3.3 Integrating the frameworks

**FIGURE 1:**
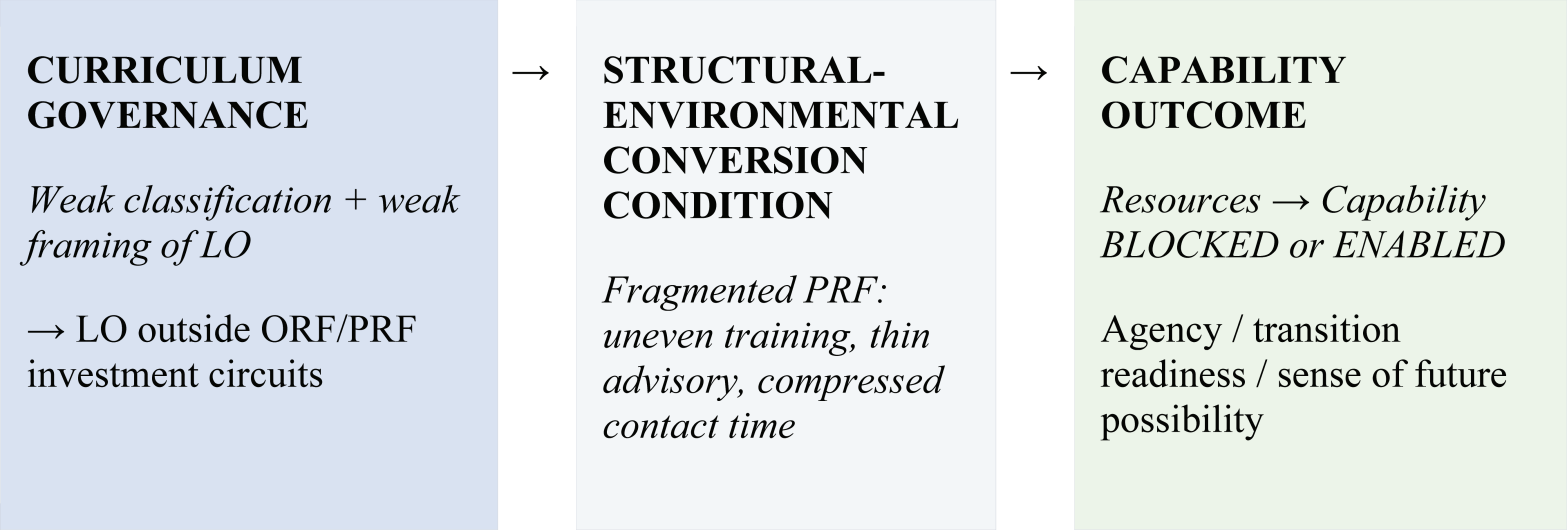
Curriculum hierarchy as a structural conversion condition: conceptual model. Arrows denote theoretical mediation relationships, not direct causal pathways. Empirical validation of the model’s components requires dedicated quantitative and mixed-methods research beyond the scope of this conceptual review.

Bringing Bernstein’s (2000) governance framework together with the Capability Approach generates a specific analytical claim. The classification and framing rules governing LO’s position within CAPS function as a structural-environmental conversion condition. They mediate whether formal access to career guidance can be converted into substantive vocational capability. Figure 1 presents the resulting model schematically.

Three implications follow from this integration. First, career guidance inequality cannot be adequately explained at the level of individual teacher competence or learner motivation; its structural determinants reside in curriculum governance. Second, “access” to career guidance is more accurately understood as a qualitative continuum than as a binary presence or absence. That continuum runs from nominal enrollment in a curriculum subject at one end to genuine capability to plan and pursue a meaningful post-school pathway at the other. Third, interventions that target service delivery without engaging the governance framework above it is unlikely to produce durable change.

## 4 Conceptual analysis: four propositions

The framework develops through four conceptual propositions, each grounded in existing South African scholarship and derived theoretically from the integration of Bernstein (2000) and Robeyns (2005). The propositions are illustratively supported by selected studies from the South African career guidance and Life Orientation literature. The analysis moves from structural conditions in Propositions 1 and 2, through assessment practices in Proposition 3, to learner-level capability outcomes in Proposition 4.

### 4.1 Proposition 1: curriculum classification stratifies career guidance infrastructure

The career guidance infrastructure in South African secondary schools is not evenly distributed; it is stratified by the level of school resourcing. This pattern is directly correlated with LO’s weak classification and its subsequent marginalization within the ORF and PRF investment circuits. The theoretical expectation, derived from Bernstein’s (2000) framework, is that subjects with weak classification will attract less of the institutional infrastructure on which substantive career guidance depends. This includes subject advisor support, specialist training, and continuing professional development. This expectation is broadly supported by the existing research.

Albien and Naidoo (2017) document, through interviews with adolescents in low-resourced township communities, how the limited and inconsistent career guidance available in such settings produces career myths and cultural stereotypes that constrain learners’ actual career options. Jonck and Swanepoel (2019), in a survey of 430 learners across seven Free State schools, found that school type (historically advantaged versus disadvantaged) had a statistically significant effect on career guidance service delivery and on the career path knowledge that learners derived from such guidance. Farao (2024) and Magano (2011) document how LO teachers in township high schools typically enter the subject without specialist preparation, with most having backgrounds in Guidance, Religious Studies, or Physical Education and receiving only short induction workshops as preparation. The findings are consistent with the theory of fragmented specialist infrastructure presented here.

The implication for capability theory is significant. Existing research indicates that learners in low-income communities articulate aspirations as complex and future-oriented as those of their wealthier peers (Fataar, 2018; Theron, 2016). The point of variations is not motivational but institutional. In instances in which the PRF for LO is fragmented, learners exhibit aspirations, which Nussbaum (2011) defines as basic capabilities. What they lack is the institutional scaffolding required to convert those aspirations into combined capabilities (Spaull and Taylor, 2015), that is, the genuine opportunity to pursue a valued vocational pathway. In such cases, the condition for structural-environmental conversion is negative. It prevents the conversion of resources into capabilities, which is necessary for effective career counseling.

### 4.2 Proposition 2: non-examined status produces institutional marginalization of LO

Proposition 2 extends Proposition 1 by specifying the governance mechanism through which the conversion condition is produced. LO is excluded from the Admission Points Score calculations used by most South African universities (Diale et al., 2014), which removes the subject from the external accountability circuits that drive institutional investment in strongly classified subjects. Bernstein”s (2000) framework predicts a self-reinforcing cycle of disinvestment in such conditions. Without external accountability, there is no systemic pressure for subject advisor appointment, regular inspection, or specialist initial teacher education. Without these, implementation quality remains uneven regardless of policy intent.

The available evidence is consistent with this prediction. Diale et al. (2014) document how LO teachers in Gauteng experience their personal and professional development as systematically under-supported, with the subject treated as a low-priority area. Diale (2016) reports, in a separate Gauteng study, that LO teachers’ career development needs remain unaddressed by the institutional structures that govern the subject. Maree (2020, Chapter 6) observes that career content is characteristically delivered as isolated “career days,” sporadic events rather than the sustained developmental engagement that effective career guidance requires.

Christie (2008) provides convergent evidence from the broader post-apartheid curriculum reform context. Policy-mandated but non-examined curriculum components consistently underperform against implementation expectations in South African schools. The explanatory unit is the structural pattern, not the individual educator. Muller (2000) makes the same point in his analysis of the politics of knowledge in South African curriculum reform: weakly bounded, integrationist subjects are systematically deprioritized, not because of ideological hostility but because of the structural logic of examination governance. Interventions that target individual teacher motivation or resource provision in isolation, without addressing the governance framework, are likely to remain surface-level adjustments rather than structural solutions.

### 4.3 Proposition 3: institutional infrastructure determines the validity of career assessment

Proposition 3 establishes a link between assessment practice and governance structure. It argues that psychometric validity in career guidance contexts is not a technical property of an instrument alone, but an institutional achievement that depends on the structural-environmental conversion conditions available in the school. The position rests on two foundational frameworks. According to Messick’s (1989) unified theory of validity, the validity of a test is not determined by the test itself, but rather by the interpretation and application of its scores. Kane’s (2013) interpretive argument model specifies, in turn, that valid score use requires adequate conditions of administration, interpretation, and application.

Cultural misalignment exacerbates this issue. The career theories most widely used in South African schools include the life-span, life-space model (Super, 1980) and the vocational personality typology (Holland, 1997). These and related frameworks are listed in Leung’s (2008) catalog of the five main Western approaches. These theories were developed against the assumption of stable labor markets, linear career trajectories, and well-differentiated occupational structures, conditions that hold for relatively few South African learners. Most navigate informal economic participation, non-standard employment, and community-embedded career trajectories (Stead, 2004; Stead and Watson, 2006; Watson et al., 2022). Constructivist and contextual approaches, including the Systems Theory Framework (Patton and McMahon, 2014), career construction theory (Savickas, 2005, 2013), and the contextual action theory of career (Young and Collin, 2004), achieve greater ecological validity in such settings precisely because they take account of the structural and social conditions that shape career trajectories.

The argument advanced here is that the cultural misalignment problem is secondary to, and exacerbated by, the institutional infrastructure problem. Maree (2020, Chapter 6) reports that narrative and life-design counseling approaches grounded in Savickas; Savickas’s (2005, 2013) career construction theory are associated with enhanced engagement and decision-making confidence. Magano (2011) and Modiba and Sefotho (2019), however, document that such contextually responsive approaches remain dependent on specialist practitioner training and adequate feedback infrastructure, conditions that are structurally constrained in weakly classified LO contexts. Despite its paradoxical nature, the outcome is instructive. In well-resourced schools, even culturally imported instruments may achieve interpretive validity through the mediation of trained practitioners with adequate feedback time. In under-resourced schools, even locally developed and culturally sensitive instruments may lose their interpretive purchase when the practitioner lacks specialist training or the structural conditions for meaningful feedback dialogue. The determinant of psychometric integrity, in other words, is not tool origin. It is the institutional conversion conditions under which the tool is used.

### 4.4 Proposition 4: fragmented conversion conditions produce differential educational preparedness

Proposition 4 integrates the preceding three by specifying the learner-level outcome. Where structural-environmental conversion conditions are negative, learners experience capability deprivation in the vocational domain. Negative conversion conditions, in this context, include fragmented professional infrastructure, compressed contact time, and epistemically misaligned tools deployed without interpretive support. The claim is not about individual deficits. It is a claim about the institutional production of constrained agency.

Bantjes and Kagee (2013) report that vocational uncertainty is significantly associated with psychological distress among South African secondary school learners, with the association most pronounced in schools that have limited career guidance infrastructure. The Capability Approach provides a theoretical explanation for the finding. When structural conversion conditions prevent resource-to-capability conversion, the resulting uncertainty is existential rather than informational. This uncertainty limits the learners’ sense of future possibility and their ability to exercise practical reason, which Nussbaum (2000) 2011 identifies as a core human capability. The same dynamic is reflected in the literature on adolescent occupational identity. Where institutional support for career exploration is fragmented, the developmental task of consolidating an occupational identity is itself impeded (Skorikov and Vondracek, 2011). Spaull and Taylor (2015) document the corresponding aspirations-attainment gap. Learners in low-quintile schools do not lack ambition; they lack the institutional conversion scaffolding that would allow them to bridge aspiration and action.

In Nussbaum’s (2011) terms, these learners possess the basic capability for vocational agency, which is to say the innate human capacity to plan and pursue a meaningful future. What they lack is the combined ability to exercise it, as the institutional conditions required to develop and realize that capacity are structurally absent. Psychology of Working Theory (Duffy et al., 2016) provides convergent theoretical support, demonstrating that structural constraints, including economic marginalization and inadequate career preparation, systematically limit vocational agency among economically marginalized populations. The argument is directly applicable to the secondary school setting in South Africa.

The inverse relationship is also evident in the literature. Where institutional scaffolding is systematically provided, including specialist-trained educators, sustained career exploration integrated longitudinally, and adequate advisory contact, the available research reports more favorable outcomes in decision-making clarity, expressed agency, and psychological wellbeing (Bantjes and Kagee, 2013; Theron, 2016). The theoretical foundation for this conclusion is provided by Walker (2006), who contends that vocational agency is a capacity that can be either institutionally supported or structurally limited. Therefore, the development or suppression of vocational agency is largely determined by the school’s structural position within the curriculum hierarchy, with implications that go far beyond career guidance into more general educational policy. Table 2 summarizes the four propositions in falsifiable form and specifies the observable outcomes that would support or disconfirm each proposition.

**TABLE 2:** Testable propositions summary: falsifiable formulations and observable outcomes.

Proposition	Falsifiable formulation (if–then)	Observable outcome supporting the proposition	Observable outcome disconfirming the proposition
P1: Classification stratifies infrastructure	If LO is weakly classified, then career guidance infrastructure (advisor ratios, specialist training rates) will be lower and more variable across quintiles than infrastructure supporting strongly classified subjects.	Quantitatively lower subject advisor allocation and trained-teacher proportions in Q1–Q3 relative to Q4–Q5 for LO; and lower for LO than for Mathematics or Physical Sciences across all quintiles.	Advisor allocation and specialist training rates for LO are comparable to those for strongly classified subjects and stable across quintiles.
P2: Non-examined status produces marginalization	If LO is excluded from external examination and admission point calculations, then it will attract lower institutional priority in school-level resource decisions than examined subjects.	Documented lower priority in school timetabling, principal ranking of institutional priorities, and staff professional development budgets for LO than for examined subjects.	LO receives priority equal to or greater than examined subjects in school-level resource allocation decisions.
P3: Infrastructure determines assessment validity	If institutional conditions (specialist training, feedback time, learner-to-counselor ratio) are absent, then the interpretive validity of career assessment scores will be compromised regardless of the instrument used.	Learner outcomes on career decision-making measures are more strongly predicted by institutional conditions than by instrument selection.	Instrument selection is the primary predictor of assessment validity outcomes, independent of institutional conditions.
P4: Fragmented conversion produces differential preparedness	If the structural-environmental conversion conditions are negative, then learners in affected schools will show measurably lower vocational agency, transition readiness, and sense of future possibility than learners in comparable schools with positive conditions.	Systematic quintile-linked differences in learner-reported vocational agency, decision-making clarity, and post-school transition indicators that track institutional conditions rather than learner aspiration.	No systematic differences on these outcomes, or differences that track learner-level rather than institutional-level variables.

In this table states each proposition in falsifiable if–then form. Observable outcomes are indicative rather than exhaustive; empirical validation would require dedicated quantitative and mixed-methods research programs across multiple schools and quintiles.

## 5 Discussion

### 5.1 Structural inequality as institutionally mediated practice

Taken together, the four propositions support a clear position. Career guidance in South African secondary schools functions as a curriculum-mediated practice embedded in structural conditions, not as an isolated informational adjunct to teaching. The mechanism of inequality is structural rather than motivational. Learners in under-resourced schools do not lack aspiration. The curriculum governance framework determines which subjects attract professional investment, and that investment, in turn, determines whether career resources can be converted into substantive vocational capabilities.

The manuscript’s contribution can now be restated with reference to its broader significance. The reconceptualization of curriculum classification and framing rules as a structural-environmental conversion condition offers a portable analytic lens for any examination-centered system in which subjects are differentially governed and resourced. The framework travels beyond the South African case because the mechanism it names, the differential capture of governance investment by strongly classified subjects, is not unique to a single national context. In this way, the paper contributes both a specific explanatory account of South African career guidance inequality and a general theoretical construct that may be productively engaged in other examination-centered settings, including those undergoing reform and those where career guidance mandates have expanded faster than the institutional infrastructure required to realize them.

This position carries significant implications for educational policy. Evaluative attention should shift from what learners are presumed to lack to what institutions structurally fail to provide. The structural reality that institutions fail to provide adequate conversion scaffolding is obscured by the standard diagnostic vocabulary, which treats learners as deficient in aspiration, ambition, or information (Mabulana, 2025). Addressing the compliance gap therefore requires more than a superficial adjustment of LO delivery. It requires engagement with the curriculum governance architecture that produces the gap in the first place. Service-delivery interventions, however well intentioned, are unlikely to produce durable change without reform of the classification and framing rules governing LO’s position within CAPS (Mabulana, 2025).

### 5.2 Toward an operational research agenda

The conceptual framework generates a specific empirical research agenda. Curriculum classification and framing rules are theorized here as structural-environmental conversion conditions, and measuring such conditions calls for the development of validated institutional indicators.

Establishing such a monitoring framework would allow the compliance gap to be operationalized as a measurable institutional phenomenon rather than an anecdotal observation. This is a prerequisite step for evidence-based policy reform. The theoretical framework advanced here provides the analytic foundation on which such a measurement agenda could rest.

### 5.3 Transferability to other examination-centered systems

The mechanism theorized here is not specific to South Africa. Comparative policy analyses indicate that examination-centered systems internationally tend to deprioritize non-examined developmental subjects, which receive fewer resources, less professional investment, and lower institutional priority (Hooley et al., 2013; OECD, 2004; Sultana, 2011; Watts and Fretwell, 2004). According to Akosah-Twumasi et al. (2018), institutional and contextual limitations that closely resemble those found in South Africa are among the structural and social factors influencing adolescents’ career decision-making in Sub-Saharan Africa. As a result, the framework is more widely applicable. It applies to any examination-centered system in which career guidance is mandated but positioned within a governance hierarchy that withholds professional investment from non-examined subjects. This includes systems undergoing examination reform, systems experiencing growing misalignment between labor market requirements and examination curricula, and systems in which career guidance has been legislatively mandated but insufficiently resourced.

## 6 Implications for policy, practice and curriculum reform

The framework generates directions for three categories of actor. Each subsection below draws directly from the four propositions and translates them into actionable implications.

### 6.1 Implication for policy

Three implications follow for national and provincial education policy actors. First, monitoring priorities need to be reclassified so that non-examined developmental subjects are visible in official data. If LO subject advisor ratios, specialist training rates, and learner-to-counselor contact hours are not routinely reported at provincial and district level, the compliance gap identified in this paper cannot be operationalized as a policy problem. Second, subject advisor allocation for LO should be standardized across school quintiles rather than left to provincial discretion, so that historically disadvantaged schools are not systematically underserved. Third, the reporting of career guidance infrastructure indicators (Table 3) should be treated as a mandatory element of school- and district-level accountability, with reporting integrated into existing curriculum monitoring frameworks.

**TABLE 3:** Operational indicators for the structural-environmental conversion condition.

Indicator	Theoretical justification	Proposed measurement approach	Empirical threshold (to be established)
LO subject advisor ratio per 10,000 secondary learners per district	Direct index of PRF investment in the subject; Bernsteinian marker of institutional recognition.	Administrative audit of provincial education department appointment records against enrollment data.	Threshold requires derivation from comparative analysis of subject-advisor ratios for strongly classified subjects across quintiles.
Proportion of LO educators with accredited specialist training in career counseling	Marker of professional infrastructure quality; connects Bernsteinian classification to Kane (2013) validity conditions.	Survey of LO educators in a stratified sample of schools, cross-checked against provincial accreditation records.	Threshold contingent on definition of accredited specialist training; comparative baseline required.
Average learner-to-counselor contact ratio (hours per learner per year)	Direct index of conversion capacity; captures whether resources are usable by particular learners in particular settings.	School-level administrative records combined with timetable audits across a representative sample.	Threshold to be established relative to minimum contact required for validity in Kane’s (2013) argument-based framework.
Frequency and duration of subject advisory school visits per annum	Marker of institutional presence within the PRF; index of ORF–PRF coupling.	Administrative records of subject advisor visits, cross-referenced against school self-reports.	Threshold to be established relative to subject advisory visit patterns for strongly classified subjects.
Institutional priority ranking of LO in school-level resource decisions	Index of ORF/PRF marginalization within the school; Bernsteinian marker of governance status.	Structured interviews with school principals; document analysis of school planning and budgeting.	Threshold to be established through comparative analysis relative to strongly classified subjects.

In this table translates the framework into a concrete measurement program. Each indicator is theoretically justified, and each requires empirical validation before deployment as a monitoring instrument. The indicators are not currently collected or reported systematically in publicly accessible South African education administrative data (Spaull and Jansen, 2019).

### 6.2 Implications for practice

Three implications follow for LO teachers, school-based support teams, and career practitioners working in resource-constrained settings. First, professional development pathways should include accredited specialist training in career counseling, recognizing that generalist LO training has been documented as insufficient to produce valid career guidance practice (Diale, 2016; Magano, 2011). Second, career content should be delivered as sustained developmental engagement across the school year rather than as episodic “career days” (Maree, 2020), so that learners can develop a coherent occupational identity over time (Skorikov and Vondracek, 2011). Third, where psychometric instruments are used, interpretive scaffolding must be built into the delivery model, including adequate feedback time and learner-to-counselor ratios that allow substantive dialogue about assessment results (Kane, 2013; Messick, 1989).

### 6.3 Implications for curriculum reform

Three implications follow for curriculum designers. First, the classification and framing of LO within CAPS should be reconsidered in light of the framework advanced here. Weak classification is not merely a design feature; it is a governance mechanism that determines investment. Where the state judges that career guidance should attract institutional weight, it must accept that some strengthening of classification and framing is required. Second, career content might be integrated into examined subject areas rather than confined to a non-examined subject, so that the accountability circuits that drive investment in examined subjects also carry career guidance provision. Third, career guidance infrastructure metrics should be included in the curriculum policy monitoring framework itself, so that curriculum reform is evaluated on the institutional conditions it produces and not only on the content it mandates.

## 7 Limitations

Several limitations circumscribe the conclusions of this conceptual review. First, the framework develops primarily through engagement with South African scholarship. While comparative references support the claim of theoretical transferability, the framework has not been empirically tested in other national contexts. Contextual application elsewhere requires adaptation of the specific institutional indicators proposed.

Second, the four propositions are theoretically derived and illustratively supported, not empirically tested within this review. The framework generates a set of testable hypotheses but confirming or disconfirming them will require dedicated empirical research, including longitudinal studies tracking the conversion efficiency of different institutional configurations across learner cohorts.

Third, the operational indicators proposed in section 4.2 have not been psychometrically validated as measurement constructs. Practitioner-to-learner ratios, accredited training proportions, and advisory contact time are theoretically grounded but illustrative benchmarks. Their operationalization as reliable institutional measures will require dedicated scale development, together with cross-contextual validation, before they can be deployed systematically.

Fourth, the review draws primarily on research addressing the secondary schooling context. The mechanisms theorized here may operate differently in primary or tertiary education, and their application to other educational levels therefore calls for caution.

Fifth, approximately one-third of the research drawn upon was published between 2020 and 2024, a period during which South African schooling was substantially disrupted by the COVID-19 pandemic. Certain studies from this time frame might be more indicative of pandemic-induced than structural baseline conditions. The severity of the institutional fragmentation patterns depicted here may be overstated in comparison to pre-pandemic norms, which should be considered when interpreting specific findings.

## 8 Conclusion

This conceptual review has advanced a theoretically distinctive framework for explaining career guidance inequality in South African secondary schools. Its principal contribution is the conceptualization of curriculum classification and framing rules as a form of structural-environmental conversion condition, an institutional configuration that mediates the transformation of career guidance resources into substantive vocational capability. This construct sits at the intersection of Bernsteinian curriculum sociology, capability-approach educational scholarship, international career guidance policy studies, and South African career psychology, and it opens each of those traditions to new lines of enquiry. The framework integrates Bernstein’s (2000) sociology of knowledge, drawing in particular on classification, framing, and the recontextualizing fields, with Sen’s (1999) Capability Approach as elaborated by Robeyns (2005), Walker and Unterhalter (2007), and Nussbaum (2000) 2011. Four conceptual propositions develop this argument and ground it in existing South African research. The first is that weak classification of LO stratifies career guidance infrastructure across school quintiles by withdrawing PRF investment from non-examined subjects. The second is that LO’s exclusion from external accountability circuits produces a self-reinforcing cycle of disinvestment, which marginalizes career guidance regardless of the formal policy mandate. The third is that institutional infrastructure, not tool origin, is the primary determinant of psychometric validity in career assessment contexts. The fourth is that these structural conditions produce differential educational preparedness outcomes that are best understood as institutionally mediated capability deprivation rather than as individual learner deficit. The central contribution of this review is to show that career guidance inequality is not primarily a deficit of learner aspiration. It is a consequence of institutional conditions that shape how resources are converted into capability. In a deeply unequal education system, career guidance can either interrupt or reinforce existing patterns of advantage and disadvantage. Which of the two it does depend not on the dedicated efforts of individual educators or the technical properties of career assessment instruments, but on the structural conditions governing the practice and, specifically, on the curriculum governance hierarchy that determines institutional investment. Addressing career guidance inequality therefore requires more than peripheral adjustment of LO delivery. It requires engagement with the governance logic that constitutes one structural mechanism through which educational inequality is institutionally reproduced.
